# Supplementary material for: Reviewing challenges in access to oral health services among the LGBTQ+ community in Indiana and Michigan: A cross-sectional, exploratory study
Source: PLoS One. 2022 Feb 25;17(2):e0264271. doi: 10.1371/journal.pone.0264271 (PMC8880834; doi:10.1371/journal.pone.0264271)
Supplement: S1 File — This is the S1 Appendix I. (DOCX) [file pone.0264271.s001.docx]

**Appendix I**

**Patient Questionnaire**

# Part I: Demographics

What is your age? _____________________

Do think of yourself as (check one)?

- Lesbian, gay, or homosexual
- Straight or heterosexual
- Bisexual
- Something else
- Don't know

What is your current gender identity? (Please select one)

- Male
- Female
- Female-to-Male (FTM)/Transgender Male/Trans Man
- Male-to-Female (MTF)/Transgender Female/Trans Woman
- Genderqueer, neither exclusively male nor female
- Additional gender category, please specify: ____
- Decline to answer

## What sex were you assigned at birth on your original birth certificate? (Check one)

- Male
- Female
- Decline to answer

Select your racial identity.

- White or Caucasian
- Black or African American
- Asian
- American Indian/Alaskan Natives
- Hawaiian / Pacific Islander
- Other: ___________
- Multiracial

## Select your ethnicity.

- Hispanic
- Non-Hispanic

Zip code of your home address: ________________

Name of your dental provider or their clinic (mention no dental provider if you do not have )__:_________________________________________

## **Part II: Please mark how you feel about the following statements.**

|  | Strongly disagree | Disagree | Neither agree nor disagree | Agree | Strongly agree |
| --- | --- | --- | --- | --- | --- |
| I think dental care is a necessity and it affects my overall health and quality of life |  |  |  |  |  |
| I am comfortable going to dental appointments. |  |  |  |  |  |
| I think that people in the LGBTQ+ community have additional obstacles accessing healthcare. |  |  |  |  |  |

**Part III: Please respond for the dental clinic you are attending**

|  | Strongly disagree | Disagree | Neither agree nor disagree | Agree | Strongly agree |
| --- | --- | --- | --- | --- | --- |
| This dental clinic has created a welcoming space for the LGBTQ+ community. |  |  |  |  |  |
| In the dental clinic there are posters/ artwork that specifically caters to the LGBTQ+ community in waiting room areas/common spaces. |  |  |  |  |  |
| The medical history forms used in this facility have a place for me to indicate my preferred pronouns. |  |  |  |  |  |
| The staff in this dental clinic treats patients in the LGBTQ+ community the same as heterosexual, non- transgender (cis gender) patients. |  |  |  |  |  |
| It takes a long time to get a dental appointment at my clinic |  |  |  |  |  |

## In the last 12 months/prior to COVID-19, did you visit your dentist regularly (at least twice a year)?

## Yes

## No

In the last 12 months/ prior to COVID-19, did you have instances where you needed to see your dentist, but could not due to financial issues?

- Yes
- No

## In the last 12 months/ prior to COVID-19, have you been treated unfairly at this dentist's office because of your sexual identity or sexual orientation?

## Yes

## No

# Part IV: Improvements

Have you ever had any experiences with your dental provider and the staff in their office that you wish had gone differently? Please explain what happened, how it happened, and how it felt to you as they related to you as a member of the LGBTQ+ community.

____________________________________________________________________________________

## What do you think could be improved about your current dental clinic to make you feel more comfortable?

## __________________________________________________________________________

# Staff Questionnaire

# Beliefs, perceptions and attitudes of dental professionals and dental clinic staff

# PART I: Demographics

1. What percent of your patient population identiﬁes as part of the LGBTQ+ community?

- <5 %
- 5-10%
- 10-25%
- 26-50 %
- 51-75%
- >75%
- Do not know

Zip code in which your organization is located: ____________

In what type of setting do you practice? (Check the one setting in which you spend the most time)

- General Dental Practice
- Specialty Practice
- Hospital Based Clinic
- Dental Service Organization
- Academic Institution
- Local Health Department
- Federally Qualiﬁed Health Center (FQHC)
- Mobile Dentistry Practice
- Other

What role do you play in your organization?

- Dentist
- Dental assistant
- Dental hygienist
- Receptionist
- Other: ______

How long have you been practicing at this organization?

- Less than a year.
- 1 -2 years
- 2-4 years
- More than 4 years

What is your age? _____________

Do you think of yourself as (check one)?

- Lesbian, gay, or homosexual
- Straight or heterosexual
- Bisexual
- Something else
- Don’t know

What is your current gender identity (check one)?

- Male
- Female
- Female-to-Male (FTM)/Transgender Male/Trans Man
- Male-to-Female (MTF)/Transgender Female/Trans Woman
- Genderqueer, neither exclusively male nor female
- Additional gender category, please specify:
- Decline to answer

What sex were you assigned at birth on your original birth certiﬁcate (check one):

- Male
- Female
- Decline to answer

Racial Identity:

- White or Caucasian
- Black or African American
- Asian
- American Indian/Alaskan Natives
- Hawaiian /Paciﬁc Islander
- Other
- Multiracial

Ethnicity:

- Hispanic
- Non-Hispanic

How do you aﬃliate with the LGBTQ+ community? (Check all that apply)

- I am a member of the LGBTQ+ community.
- I have a close friend or family member who is part of the LGBTQ+ community.
- I am an ally
- I have no aﬃliation to the LGBTQ+ community.

# PART II: Belief System

|  | Strongly disagree | Disagree | Neither agree nor disagree | Agree | Strongly agree |
| --- | --- | --- | --- | --- | --- |
| I feel comfortable providing care for patients who are part of the LGBTQ+ community. |  |  |  |  |  |
| I feel comfortable working with coworkers who are part of the LGBTQ+ population. |  |  |  |  |  |
| I feel it is my responsibility to care for patients who are part of the LGBTQ+ community. |  |  |  |  |  |
| I think sexual/ gender minorities have less access to seek healthcare services. |  |  |  |  |  |
| I am willing to learn and utilize resources to improve access to care for patients who are part of the LGBTQ+ community. |  |  |  |  |  |
| I believe I have undergone adequate training regarding cultural competency and the LGBTQ+ community. |  |  |  |  |  |
| I am aware of the changes one’s body goes through when on hormone therapy and its eﬀects on overall health and how it may relate to oral health. |  |  |  |  |  |

**PART III: What currently exists in your clinic?**

|  | **True** | **False** |
| --- | --- | --- |
| In this clinic, we have created a welcoming space for the LGBTQ+ community. |  |  |
| There are posters/artwork that specifically caters to the LGBTQ+ community in the waiting room areas/common spaces. |  |  |
| The medical history forms have a place for the patient to indicate their preferred pronouns. |  |  |
| The staff treats patients who are part of the LGBTQ+ community. the same as heterosexual, non-transgender (cisgender) patients. |  |  |
| I know who to ask if I have a question about LGBTQ+ care. |  |  |

**PART IV: Improving Patient's Quality of Care.** Please respond to the following statements.

|  | Strongly disagree | Disagree | Neither agree nor disagree | Agree | Strongly agree |
| --- | --- | --- | --- | --- | --- |
| Patients should be able to indicate their preferred pronouns on patient registration forms. |  |  |  |  |  |
| Providers should attend continuing education/training courses on the care of LGBTQ+ patients. |  |  |  |  |  |
| Hiring employees from the LGBTQ+ community improves patients’ quality of care. |  |  |  |  |  |

# PART V: Open ended

Are there any experiences you would like to share with us about your experiences as they relate to working with patients who are part of the LGBTQ+ community? _________________________________________________
