# Supplementary material for: Reviewing challenges in access to oral health services among the LGBTQ+ community in Indiana and Michigan: A cross-sectional, exploratory study
Source: PLoS One. 2022 Feb 25;17(2):e0264271. doi: 10.1371/journal.pone.0264271 (PMC8880834; doi:10.1371/journal.pone.0264271)
Supplement: Attachment — Submitted filename: rebuttal.docx [file pone.0264271.s003.docx]

2/1/2022

PONE-D-21-36125

Reviewing Challenges in Access to Oral Health Services Among the LGBTQ+ Community in Indiana and Michigan: A Cross-Sectional, Exploratory Study

Dear Editors,

We would like to thank the reviewers for their valuable time and effort in reviewing our manuscript. We have addressed all the comments and made edits in the blinded manuscript suing track changes as recommended.

The manuscript has been formatted to comply with the PLOSone style. The funding information has been removed from Acknowledgments section and updated in the funding statement. Study’s minimal underlying data set is uploaded as Supporting Information file and references checked.

Please find our **responses** to the comments and suggestions below, for additional clarification.

Reviewer #1: Tharp and colleagues reported on oral healthcare providers (OHP)’s perception and practice toward LGBTQ+ patients, and, conversely, the experiences of LGBTQ+ patients in oral healthcare settings. The manuscript is overall clear and well written. Methods are sufficiently sound. The topic is interesting, since policies towards LGBTQ+ community can ultimately enhance their healthcare access and health status. I only have some minor comments to the authors.
The study was conducted in Indiana and Michigan. A brief summary of the overall LGBTQ+ attitude in these states should be added to put results into different contexts.

**Response:** Thanks for the excellent suggestion, we included this information under discussion section of the manuscript.

“The survey for patients was distributed via the patient portal at these clinics”. How many participants were potentially reached? How many actually commenced on the survey? And how many screened negative? I believe this information

**Response:** It’s not possible to get this information as each of the participating clinics because of privacy laws, but it was distributed to all the patients attending those clinics. The total number of patients who commenced was the total number of patient responses we received

Was educational status collected? This is quite relevant variable, also to be considered ins statistical models.

**Response:** we understand the usefulness of collecting this information but unfortunately, we did not ask for this in our survey, it has been included as a limitation of the study.

In the discussion, authors should mention that poor attitude towards LGBTQ+ individuals can affect resource utilizations and LGBTs health status (10.1016/j.msard.2017.02.001), also with implications for oral health.

**Response:** Thanks for the excellent suggestion, we included this information under discussion section of the manuscript.

Thank you again for your valuable time and effort in reviewing our manuscript.

I remain available for any questions.

Dr. Anubhuti Shukla
